# The effects of training and sex on cardiac adaptation in elite rowers across a competitive season

**DOI:** 10.1007/s00421-025-05897-w

**Published:** 2025-07-25

**Authors:** Sarah R. Henley-Martin, Carly J. Brade, Hugh Riddell, Sophie P. Watts, Andrew J. Maiorana, Julie J. Collis, Daniel J. Green, Louise H. Naylor, Martyn J. Binnie, Angela L. Spence

**Affiliations:** 1 Exercise Science Discipline, Curtin School of Allied Health, Curtin University, GPO U1987, Perth, WA 6845 Australia; 2 School of Human Sciences (Exercise and Sport Sciences), The University of Western Australia, Crawley, Australia; 3 Western Australian Institute of Sport, Mt Claremont, Australia; 4 Department of Allied Health, Fiona Stanley Hospital, Murdoch, Australia

**Keywords:** Speckle-tracking echocardiography, Female athlete, Transthoracic echocardiography, Cardiac hypertrophy

## Abstract

**Purpose:**

Exercise-induced cardiac adaptation is well-studied in male athletes. However, evidence for longitudinal adaptation, particularly in females, is limited. This study compared cardiac adaptation between elite female and male rowers across a competitive season.

**Methods:**

Ten females (19 ± 0.9 years) and 11 males (20 ± 1.7 years) were assessed across 21 weeks at early (ES), mid- (MS), and late season (LS). Echocardiography (2D, 3D, strain), peak oxygen consumption (V̇O_2_peak), weekly training volume (sessions) and intensity (minutes in heart rate zones) were documented. Bayesian two-way repeated measures ANOVA assessed sex and training effects.

**Results:**

Training volume was comparable between sexes; however, females spent less time at maximal heart rate intensity. An interaction effect for V̇O_2_peak demonstrated the highest value at LS in females (55.8 ± 1.6 mL kg min^−1^) and MS in males (64.1 ± 1.6 mL kg min^−1^). Females had smaller left ventricular (LV) 2D mass and volume with no training-induced response in either sex. A sex-specific interaction was observed for 3D LV mass: females peaked at MS (221.2 ± 20.6 g) compared to males at LS (301.9 ± 20.9 g). Females also had smaller LV diameter, wall thickness and right heart dimensions. Across the season, most females (67%) exhibited eccentric hypertrophy, whilst males (89%) showed concentric hypertrophy. However, classifications varied throughout the season. No sex or training effects were observed for strain.

**Conclusion:**

Whilst sex strongly influences cardiac morphology in elite rowers, sex-specific adaptation in 3D LV mass and LV geometry changes emphasise cardiac plasticity with training in athletes.

**Supplementary Information:**

The online version contains supplementary material available at 10.1007/s00421-025-05897-w.

## Introduction

‘Athlete’s heart’ describes the observable cardiac morphological differences in highly trained individuals as a result of increased physiological demands caused by repetitive loading (Bryde et al. 2021; D’Ascenzi et al. 2020; Pelliccia and Adami 2017; Weiner et al. 2015). This phenomenon is well-documented in male, and to a lesser extent, female athletes, with cardiac morphological parameters in male athletes often exceeding the ‘normal’ limits considered by the American Society of Echocardiography (Churchill et al. 2021). This paucity of female-specific data precludes clinical differentiation between physiological adaptation and pathological dysfunction, necessitating sex-specific analyses to guide individualised diagnosis and management of female athletes (Patel et al. 2022). Imaging approaches, such as three-dimensional echocardiography (3D) and speckle-tracking echocardiography (STE), enable highly sensitive discrimination of cardiac remodelling and myocardial mechanics respectively (D’Andrea et al. 2021). Together, this allows for a comprehensive appreciation of exercise-mediated cardiac adaptations with continued training in athletes (Wasfy et al. 2019). Studies in previously untrained participants demonstrate morphological changes, such as increased chamber size and volume, occur quickly with training stimuli, particularly in the first 3 months (Howden et al. 2015). With chronic high volumes of training, evident in sports such as rowing, the heart remains malleable and can result in further wall thickening and regression of left ventricular (LV) twist mechanics (Kleinnibbelink et al. 2021; Weiner et al. 2015) which is directly influenced by training duration and type (Castillo et al. 2023; D’Ascenzi et al. 2020). The interrelationship between training mode, duration and intensity effects on cardiac remodelling and performance, in female athletes especially, remains largely unexplored.

Sex also impacts cardiac phenotype (Bryde et al. 2021; D’Ascenzi et al. 2020; St. Pierre et al. 2022) whereby the female cardiovascular system differs in both morphology and function compared to males, exhibiting smaller absolute cardiac dimensions yet higher ejection fractions (Nio et al. 2015; Pelliccia and Adami 2017; St. Pierre et al. 2022). Female hearts typically have a smaller internal diameter for the same LV length in males, resulting in a more ellipsoid geometry at end diastole compared to males (Pelliccia and Adami 2017; Williams et al. 2017). As a result, LV mechanics in females may be influenced by LV geometry, thereby contributing to differences in response to acute cardiovascular stress (Williams et al. 2017). For example, female hearts appear to be more reliant on contractility and ventricular twisting to maintain stroke volume under acute loading conditions (Williams et al. 2016). This results in a greater degree of apical rotation when compared to their male counterparts. This sex-specific response to exercise has also been observed when previously untrained males and females engaged in exercise interventions (Howden et al. 2015; Marsh et al. 2021). Further, females have been shown to adapt by developing eccentric hypertrophy (concomitant increase in increased ventricular mass and volume), whereas up to 15% of male endurance athletes develop concentric LV hypertrophy (increased cardiac mass with little to no change in volume; (Finocchiaro et al. 2017). It remains unclear whether LV geometry changes with training in athletes or whether any training-induced adaptations are mediated by sex.

Females are significantly underrepresented in sport and exercise science research (Cowley et al. 2021). Studying female physiology is complex, primarily due to the variation of ovarian hormone profiles between and within individuals with a menstrual cycle or those using hormonal contraception (D’Souza et al. 2023; Elliott-Sale et al. 2021). When females are included in research studies, menstrual status and hormone verification are often unreported or sex-disaggregated data are not presented (Elliott-Sale et al. 2021). Furthermore, oestrogen receptors are found throughout the cardiovascular system, including cardiac tissue, underpinning the hypothesis that oestrogen is an important regulator of cardiovascular function and key contributor to observable sex differences (Nio et al. 2015; Pelliccia and Adami 2017). With known differences within the cardiovascular system, a sex-specific approach in assessing exercise-induced cardiac adaptation is warranted as data derived from male athletes cannot be directly applied to female athletes.

Whilst observable physiological adaptations of the human cardiovascular system occur due to high training load (Weiner et al. 2015), specific changes across a competitive training season in an elite cohort, and comparison between the sexes, are yet to be fully explored. Therefore, this observational study aims to temporally evaluate exercise-induced cardiac adaptation in elite, highly trained females compared to male athletes, across a 21-week competitive rowing season, specifically early in the season (early season), at the end of the general preparatory phase (mid-season) and at the conclusion of the season (late season), prior to a major competition.

## Materials and methods

Curtin University Human Research Ethics Committee approved this study (HRE2020-0510), and written, informed consent was given by all participants prior to participation in the study.

### Participants

Highly trained, elite young rowers were recruited from the Western Australian Institute of Sport performance pathway programme. Athletes were classified according to the Participant Classification Framework (McKay et al. 2022) with *n* = 16 state-level representatives (Tier 3 Highly Trained/National Level; female *n* = 8) and *n* = 5 on the national junior team (Tier 4 Elite/International Level; female *n* = 2). Training age was calculated as the time spent in sport-specific training at or above Tier 3 until enrolment in the study. Pre-enrolment medical screening was used to assess the suitability of the study to the participants. This included a questionnaire for females relating to previous history of menstrual status including self-reported menstrual cycle characteristics (e.g. length of cycle, length of menstruation), symptoms and use of hormonal contraception (including type, duration, formulation). Due to the observational nature of the study, female athletes using and not using hormonal contraception were included. Prior to study enrolment, we confirmed consistent (> 3 month) use of either the same type of hormonal contraception or lack of hormonal contraception. Participants were also asked to maintain this for the duration of the study unless directed otherwise by their medical professional. Menstrual status was not controlled for at measurement time points, although was rigorously documented using a combination of subjective and objective measures, according to current best-practice guidelines (Elliott-Sale et al. 2021) and reported in the results section.

### Study overview

**Fig. 1:**
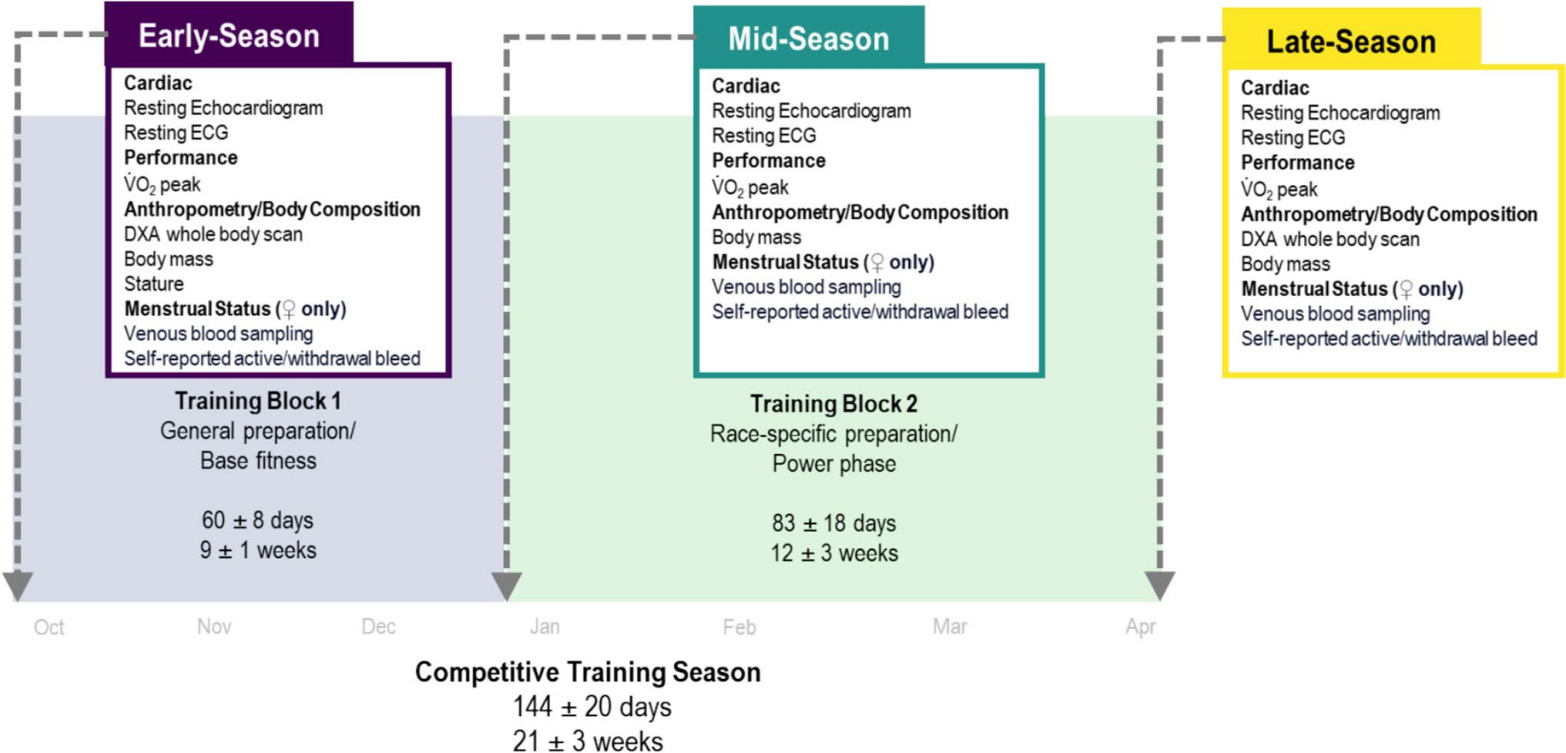
Overview of study conducted during a competitive season for elite female (*n* = 10) and male (*n* = 11) rowers, including timeline of season, number of days (mean ± standard deviation) between data collection time points (Early Season, Mid-season, Late Season), data collected at each time point and specific training goal during each training block (Training Block 1 and Training Block 2)

Athletes were observed across a competitive rowing training season (21 weeks) with testing protocols taking place on three separate occasions, aligned with key time points in the training season; early season (ES), mid-season (MS), and late season (LS; before major competition). The training season was divided into 2 specific training blocks, Block 1 (ES-MS, general preparation, base strength and overall fitness phase) and Block 2 (MS-LS, race-specific preparation and power phase). The testing protocol consisted of a resting cardiac assessment, an oxygen consumption (V̇O_2_peak) assessment and body composition and anthropometric assessments as described in each section detailed below (Fig. 1).

### Cardiac measures

Transthoracic echocardiographic measures were taken at rest by the same experienced sonographer (JC) using a commercially available ultrasound system (EPIQ-7, Koninklijke Philips N.V., Andover, MA) using an X5-1xMATRIX array (1–5 MHz) transducer. A 12-lead electrocardiogram (ECG; Nassif Associates Inc., NY, USA) was obtained to measure resting heart rate.

Measurements were obtained following the American Society of Echocardiography guidelines (Baggish et al. 2020; Mitchell et al. 2019; Lang et al. 2015). Cardiac morphology and function were measured utilising the following parameters: LV internal dimension at end diastole (LVIDd), end systole (LVIDs), LV end-diastolic posterior wall (LVPWd) and interventricular septum (IVSd), relative wall thickness (RWT), LV end-diastolic volume (LV EDV), LV end-systolic volume (LV ESV), stoke volume (SV), left atrial (LA) area, LA volume, LA end-systolic volume (LA ESV), right atrial (RA) area, RA end-systolic volume (RA ESV), right ventricular (RV) diameters specifically basal and mid-cavity, tricuspid annular plane systolic excursion (TAPSE), peak systolic velocity of the tricuspid annulus (RV S′), peak early mitral inflow velocity (*E*), mitral annular early diastolic velocity (*e′*), and the ratio between them (*E*/*e′*) (Baggish et al. 2020; Mitchell et al. 2019; Lang et al. 2015). Ejection fraction was calculated using the biplane method, the cubed equation was used to calculate left ventricular mass (LVM) (Mitchell et al. 2019). Body surface area (BSA) and dual-energy absorptiometry (DXA) derived lean body mass (LBM), as described below in Body Composition and Anthropometry section, were used to index values to body size (indexed to body surface area, iBSA; indexed to lean body mass, iLBM). The same sonographer (JJC) analysed all images using specialised software (TOMTEC imaging systems GMBH, Unterschleissheim, Germany). Intra-rater reliability was measured by a blinded re-analysis of 12 echocardiograms chosen by a random number generator and assessed with an Intraclass Correlation Coefficient (0.851, indicating excellent reliability).

Two-dimensional (2D) speckle-tracking echocardiography (2DSTE) was utilised to measure global longitudinal strain (GLS) from the average of the apical 3-chamber, 2-chamber and 4-chamber views (Negishi et al. 2015). Three-dimensional (3D) images from the apical window were analysed for twist, torsion, 3D LV EDV (iBSA), 3D LVM (iBSA and iLBM) and LV strain (3D GLS, 3D global strain, 3D global circumferential strain [GCS] and 3D global radial strain [GRS]) (Muraru et al. 2018).

A 4-tier classification system was used to assess LV geometry and cardiac hypertrophy phenotype. Left ventricular hypertrophy was determined using sex-specific thresholds for LVM iBSA (≥ 96 g/m^2^ for females; ≥ 116 g/m^2^ for males) relative to concentricity index (LVM/EDV; ≥ 8.1 g/mL^0.67^ females; ≥ 9.1 g/mL^0.67^ males) to classify athletes as having normal geometry, eccentric LV hypertrophy, concentric LV hypertrophy or concentric remodelling. Athletes with either eccentric or concentric LV hypertrophy were further classified as having dilated hypertrophy using threshold of ≥ 76 mL/m^2^ (both sexes) for LV EDV iBSA (Khouri et al. 2010).

### Performance measures

Athletes completed a graded exercise test on a rowing ergometer (Concept II, Morrisville, NC) with expired breath collected and analysed via a metabolic gas analysis system (TrueOne 2400, Parvo Medics, Inc., UT, USA) for the determination of V̇O_2_peak. As per the Rowing Australia Seven Step Rowing Protocol, the test included seven stages, increasing in intensity, based on previous 2 km time trial individual results. Workload was assigned for each 4-min stage, with the final stage being a maximal effort. Heart rate and V̇O_2_ were recorded throughout the test and blood lactate concentration was recorded in the 1-min break between stages.

### Training volume and intensity

Training for all participants was tracked throughout the 21-week season. Number and type of sessions per week (conditioning or resistance session) were recorded to monitor training volume. Training intensity was recorded for all conditioning sessions, including on-water, ergometer and cross-training through heart rate (Garmin Forerunner 735XT, Garmin International, Inc., USA) and stored on TrainingPeaks (TrainingPeaks, Louisville, KY) where it was later extracted for further summaries and analysis. Heart rate was divided using a five-zone model, into the following standardised training intensity zones; T1: between 50% of V̇O_2_peak and the midway point between 50% V̇O_2_peak and lactate threshold 1, T2: between the top of T1 and lactate threshold 1, T3: between lactate threshold 1 and 95% of lactate threshold 2, T4: between 95% and 102% lactate threshold 2, T5: above lactate threshold 2 (Watts et al. 2022). Time (minutes) spent in each zone was recorded. Lactate thresholds 1 and 2 were based on the most recent V̇O_2_peak test and determined using the modified *D*_max_ method.

### Body composition and anthropometry

Body mass (kg) and standing stretch stature (cm) were assessed by the same International Society for the Advancement of Kinathropometry accredited researcher (SHM) using standard anthropometric techniques and used to determine BSA (Mosteller equation; (square root ((height/mass)*3600)). Participants underwent a whole-body DXA (Lunar Prodigy, GE Medical Systems, Madison, WI, USA) at ES and LS only, which was used to assess body composition including total lean mass (%) and total body fat percentage (%).

### Menstrual status

Females completed menstrual status tracking throughout the duration of the study, starting one week before the first testing session. This was achieved via weekly self-report with the use of a bespoke questionnaire collected on paper and stored in Excel which asked participants to log start/end dates of their menstrual period or withdrawal bleed, used to determine cycle length and number of menstrual periods or withdrawal bleeds.

A venous blood sample (serum) was collected from the antecubital vein of female participants during each testing visit, which was analysed offsite (PathWest Laboratory Medicine, Nedlands, Western Australia) for hormone concentration (oestradiol (E2, pmol/L), progesterone (P4, nmol/L) and testosterone (T, nmol/L)). This, along with cycle tracking, was used to verify cycle phase in naturally cycling participants.

### Statistical analysis

Statistical analyses were performed using JASP (Version 0.19.1.0 University of Amsterdam, The Netherlands). A Bayesian *t *test was used to assess sex differences for age, training age and height at the early-season time point. A Bayesian two-way repeated measures ANOVA was used to determine the effect of training on dependent variables as well as between athlete groups (sex). The best model for each variable is reported in text with the Bayes factor for all models available in the supplementary material. Bayes factor (BF_10_) was interpreted with the following strength of evidence: < 1 supporting the null hypothesis, 1–3 marginal/anecdotal (i.e. insufficient evidence to conclude an effect), 3–10 moderate, 10–30 strong, 30–100 very strong and > 100 extreme evidence in favour of a meaningful difference/effect (Faulkenberry et al. 2020).

Bayesian statistics allows for a more straightforward interpretation than traditional frequentist *p* values and is not dependent on large sample sizes or underlying assumptions about the distribution of data, thus offering both theoretical and practical advantages for the analysis of the current dataset. Instead of *p* values, model comparisons can be made using BF_10_ to evaluate the magnitude of evidence supporting alternative models (i.e. those including one or more predictor variables) over the null (i.e. a model with no predictors, which inherently assumes study variables have no effect on the outcome). Bayes factor greater than 1 indicates evidence supporting the alternative model, whilst values less than 1 indicate evidence supporting the null model (Faulkenberry et al. 2020). Credibility intervals given by Bayesian analysis are interpreted in the same way as confidence intervals, which are the range within which the actual parameter value is likely to fall with 95% certainty (Faulkenberry et al. 2020). If the mean of the test group does not occur in the credibility intervals of the comparator group, we can conclude, with 95% certainty, that the groups are distinct, indicating that a parameter’s effect (i.e. sex, training or their interaction) is meaningful.

## Results

Twenty-two athletes were initially recruited for this study, but one female participant was injured after early-season testing which resulted in her withdrawing from the study, leaving a final sample of ten female athletes (19 ± 0.9 years, [18.5-19.8]) and eleven male athletes (20 ± 1.7 years, [19.3–21.5]). Participants’ demographical data is presented in Table 1. Differences in age and training age between male and female participants were negligible (both BF_10_ = 1.7), whereas males were taller than females (188.3 ± 6.9 cm, [183.7–193.0] vs. 177.9 ± 5.9 cm, [173.7–182.1]) with strong evidence for this sex difference (BF_10_ = 22.0). Very strong evidence for a sex difference favouring males was observed for anthropometric variables including body mass (BF_10_ = 44.6), BSA (BF_10_ = 33.4) and LBM (BF_10_ = 44.2) whilst total body fat was greater in females (BF_10_ = 32.4). Resting heart rate decreased throughout the season, irrespective of sex, supported with moderate evidence (BF_10_ = 7.9). There was no meaningful effect found for maximum heart rate (BF_10_ = 0.6).

**Table 1:** Participant characteristics, cardiorespiratory (12-lead ECG determined heart rate), performance measures (peak exercise heart rate and oxygen consumption) and dual-energy absorptiometry derived anthropometric measures (mean ± standard deviation [95% credible interval]) of elite female (*n* = 10) and male (*n* = 11) rowers across a competitive season (21 weeks), measured at three time points: early, mid- and late season. The best model to explain the data from a Bayesian repeated measures of analysis and the Bayes factor (BF_10_) are reported

Dependent variable	Female athletes (*n* = 10)			Male athletes (*n* = 11)			Best model	BF_10_
	Early	Mid	Late	Early	Mid	Late		
Age (years)	19 ± 0.9 [18.5, 19.8]	–	–	20 ± 1.7 [19.3, 21.5]	–	–	–	1.7
Training age (years)	2.0 ± 1.2 [1.1, 2.9]	–	–	3.5 ± 2.0 [2.2, 4.9]	–	–	–	1.7
Height (cm)	177.9 ± 5.9 [173.7, 182.1]	–	–	188.3 ± 6.9 [183.7, 193.0]	–	–	–	22.0
Body mass (kg)	71.3 ± 6.3 [66.8, 75.8]	71.7 ± 6.7 66.9, 76.6]	71.4 ± 7.0 [66.4, 76.4]	89.4 ± 10.2 [82.5, 96.2]	89.9 ± 10.6 [82.8, 97.0]	90.0 ± 10.2 [83.2, 96.9]	Sex	44.6
BSA (m^2^)	1.9 ± 0.1 [1.8, 2.0]	1.9 ± 0.1 [1.8, 2.0]	1.9 ± 0.1 [1.8, 2.0]	2.2 ± 0.2 [2.1, 2.3]	2.2 ± 0.2 [2.1, 2.3]	2.2 ± 0.2 [2.1, 2.3]	Sex	33.4
Total body fat (%)	22.0 ± 4.3 [18.9, 25.1]	–	22.5 ± 3.8 [19.8, 25.3]	15.8 ± 3.4 [13.4, 18.3]	–	15.6 ± 2.8 [13.7, 17.9]	Sex	32.4
Lean body mass (%)	75.0 ± 4.1 [72.1, 77.9]	–	74.4 ± 3.5 [71.9, 76.8]	80.8 ± 3.0 [78.7, 83.0]	–	80.9 ± 2.7 [78.9, 82.9]	Sex	44.2
Resting HR (bpm)	54.3 ± 7.5 [48.9, 59.7]	51.5 ± 9.0 [45.1, 57.9]	51.1 ± 6.5 [46.4, 55.8]	51.7 ± 7.2 [46.1, 57.2]	46.6 ± 5.2 [45.1, 57.9]	47.0 ± 4.9 [43.2, 50.8]	Training	7.9
Maximum HR (bpm)	200.3 ± 6.6 [194.7, 205.6]	197.3 ± 6.0 [192.2, 202.3]	197.9 ± 6.4 [192.5, 203.2]	198.7 ± 7.2 [193.1, 204.2]	198.6 ± 5.9 [194.0, 203.1]	198.9 ± 7.9 [192.8, 205.0]	Training	0.6
V̇O_2_peak (L min^−1^)	3.7 ± 0.3 [3.5, 4.0]	3.8 ± 0.3 [3.6, 4.1]	3.9 ± 0.2 [3.7, 4.1]	5.3 ± 0.4 [5.0, 5.6]	5.5 ± 0.3 [5.2, 5.8]	5.4 ± 0.4 [5.1, 5.8]	Training + sex	1.39 × 10^6^
V̇O_2_peak (mL kg min^−1^)	54.3 ± 1.4 [51.1, 58.0]	55.3 ± 1.6 [52.2, 59.0]	55.8 ± 1.6 [52.8, 59.6]	62.4 ± 1.6 [58.5, 65.4]	64.1 ± 1.6 [60.3, 67.2]	63.1 ± 1.6 [59.3, 66.1]	Interaction	1258.2

Variables measured only at early season were assessed using Bayesian *t* test

Best model: the repeated measures analysis of variance that best explains the data. Sex indicates a difference between males and females, training indicates a difference across the training season, Training + sex indicates independent effects of both sex and training, interaction refers to an interaction between sex + training + sex × training

BF_10_: Level of Evidence 0–1: no effect, 1–3: anecdotal, 3–10: moderate, 10–30: strong, 30–100: very strong, > 100: extreme

*Early* early season, *Mid* mid-season, *Late* late season, *BSA* body surface area, *BPM* beats per minute, *V̇O*_*2*_*peak* peak oxygen consumption, *HR* heart rate

Absolute V̇O_2_peak had overwhelming supporting evidence (BF_10_ = 1.39 × 10^6^) to demonstrate differences between sexes and with training. Specifically, males had larger V̇O_2_peak compared to females across all time points, and V̇O_2_peak was the lowest for all athletes at ES. However, an extremely strong interaction effect was evident for V̇O_2_peak expressed relative to body mass (BF_10_ = 1258.2). Again, males had consistently higher relative V̇O_2_peak compared to females at all time points. Relative V̇O_2_peak increased following training Block 1 (ES to MS) in both sexes, which continued to increase after training Block 2 (MS to LS) in females only, reaching the highest value at LS (55.8 ± 1.6 mL.kg.min^−1^). Males however experienced a decrease in relative V̇O_2_peak after training Block 2, with the highest value of 64.1 ± 1.6 mL.kg.min^−1^ recorded at MS.

### Cardiac measures

All 2D- and 3D-echocardiographic-derived cardiac data are presented in Table 2. We observed very strong evidence for the effect of sex on cardiac morphological characteristics across multiple indicator variables (all BF_10_ > 80). Specifically, there was extremely strong evidence supporting sex as a predictor of 2D LVM, with greater LVM observed in males compared to females across all time points (BF_10_ = 15724.5), which persisted when indexing to BSA (BF_10_ = 772.0). However, when indexed to LBM, the strength of the evidence was reduced to moderate (BF_10_ = 5.2).

**Table 2:** Echocardiographic-derived variables (mean ± standard deviation [95% credible interval]) in elite female (*n* = 10) and male (*n* = 11) rowers across a competitive season (21 weeks), measured at three time points: early, mid- and late season. The best model to explain the data from a Bayesian repeated measures of analysis and the Bayes factor (BF_10_) are reported

	Female athletes (*n* = 10)			Male athletes (*n* = 11)			Best model	BF_10_
	Early	Mid	Late	Early	Mid	Late		
LV Mass (g)	184.1 ± 23.2 [167.5, 200.7]	191.7 ± 28.7 [171.1, 212.2]	190.9 ± 20.7 [176.1, 205.7]	302.8 ± 35.7 [278.8, 326.7]	304.9 ± 55.4 [267.7, 342.1]	319.0 ± 57.8 [280.1, 357.8]	Sex	15,724.5
LV Mass iBSA (g/m^2^)	98.1 ± 12.9 [88.9, 107.3]	102.0 ± 15.2 [91.1, 112.8]	102.3 ± 12.0 [93.7, 110.8]	140.0 ± 16.5 [128.8, 151.1]	141.1 ± 23.4 [125.4, 156.9]	146.8 ± 24.7 [130.2, 163.3]	Sex	772.0
LV mass iLBM (g/kg)	3.5 ± 0.5 [3.1, 3.8]	–	3.6 ± 0.4 [3.3, 3.9]	4.1 ± 0.5 [3.8, 4.5]	–	4.3 ± 0.8 [3.7, 4.9]	Sex	5.2
LV EDV (mL)	112.3 ± 14.6 [101.9, 122.8]	111.2 ± 10.0 [104.0, 118.3]	112.0 ± 16.7 [100.1, 123.9]	150.8 ± 29.7 130.9, 122.8]	159.7 ± 29.5 [139.9, 179.5]	160.4 ± 30.2 [140.1, 180.1]	Sex	105.6
LV EDV iBSA (mL/m^2^)	71.3 ± 6.3 [66.8, 75.8]	70.1 ± 5.3 [66.4, 73.9]	70.6 ± 7.4 [65.3, 75.9]	75.0 ± 8.0 [69.6, 80.4]	76.9 ± 8.9 [71.0, 82.9]	76.7 ± 9.5 [70.3, 83.1]	Sex	3.4
LV ESV (mL)	36.5 ± 4.8 [33.0, 40.0]	40.8 ± 5.1 [37.2, 44.5]	40.8 ± 5.1 [37.2, 44.5]	57.2 ± 14.1 [47.7, 66.6]	63.9 ± 14.4 [54.3, 73.6]	60.1 ± 14.6 [50.3, 69.9]	Training + sex	536.9
SV (mL)	7 5.9 ± 12.0 [ 67.3, 84.5]	70.3 ± 9.5 [63.6, 77.1]	71.1 ± 11.6 [62.8, 79.4]	93.7 ± 16.6 [82.6, 104.8]	95.8 ± 19.6 [82.6, 108.9]	100.3 ± 18.8 [87.7, 113.0]	Sex	54.0
Ejection fraction (%)	67.3 ± 3.8 [64.6, 70.1]	63.1 ± 4.8 [59.6, 66.6]	63.6 ± 5.5 [59.7, 67.6]	62.3 ± 2.8 [60.5, 64.2]	60.0 ± 5.0 [56.6, 63.3]	62.7 ± 4.7 [59.5, 65.8]	Training + sex	2.8
Concentricity Index (g/mL^0.67^)	6.9 ± 0.6 [6.4, 7.3]	7.3 ± 1.0 [6.5, 8.0]	7.3 ± 0.8 [6.7, 7.8]	10.0 ± 0.8 [9.5, 10.6]	9.9 ± 1.4 [9.0, 10.9]	10.3 ± 1.4 [9.4, 11.2]	Sex	17,683.5
LVIDd (mm)	52.7 ± 2.4 [51.0, 54.4]	52.4 ± 1.8 [51.1, 53.7]	52.4 ± 2.4 [50.7, 54.1]	57.3 ± 2.6 [55.6, 59.0]	57.9 ± 2.9 [55.9, 60.0]	57.9 ± 3.2 [55.8, 60.1]	Sex	129.9
LVIDd iBSA (mm/m^2^)	28.2 ± 1.6 [27.0, 29.3]	27.9 ± 1.6 [26.8, 29.0]	28.0 ± 1.7 [26.7, 29.2]	26.6 ± 2.1 [25.2, 28.0]	26.8 ± 2.1 [25.4, 28.2]	26.8 ± 2.1 [25.4, 28.2]	Sex	0.7
LVIDs (mm)	34.4 ± 2.1 [32.8, 35.9]	35.4 ± 1.5 [34.3, 36.5]	35.6 ± 2.1 [34.1, 37.1]	37.9 ± 2.9 [35.9, 39.9]	39.9 ± 2.6 [38.1, 41.6]	39.2 ± 3.2 [37.0, 41.3]	Training + sex	619.9
IVSd (mm)	9.2 ± 0.9 [8.5, 9.8]	9.7 ± 1.0 [9.0, 10.4]	9.5 ± 0.8 [9.0, 10.1]	12.0 ± 0.8 [11.5, 12.6]	12.2 ± 1.3 [11.3, 13.0]	12.5 ± 1.2 [11.7, 13.4]	Training + sex	11,044.2
LVPWd (mm)	9.7 ± 0.7 [9.2, 10.2]	9.9 ± 1.4 [8.9, 10.9]	10.1 ± 1.0 [9.4, 10.8]	12.7 ± 1.0 [12.0, 13.4]	12.3 ± 1.4 [11.4, 13.3]	12.7 ± 1.4 [11.8, 13.7]	Sex	1833.0
RWT (cm)	0.36 ± 0.03 [0.34, 0.9]	0.38 ± 0.06 [0.33, 0.42]	0.39 ± 0.05 [0.36, 0.42]	0.45 ± 0.05 [0.42, 0.48]	0.42 ± 0.05 [0.39, 0.46]	0.44 ± 0.05 [0.40, 0.47]	Sex	11.2
LA area (cm^2^)	21.3 ± 2.5 [19.5, 23.1]	21.6 ± 2.5 [19.8, 23.4]	22.0 ± 4.2 [19.0, 25.0]	24.9 ± 3.0 [23.0, 26.9]	21.6 ± 3.4 [23.7, 28.3]	25.8 ± 4.5 [22.8, 28.8]	Sex	15.7
LA volume (mL)	67.6 ± 16.8 [55.6, 79.6]	65.8 ± 12.8 [56.6, 75.0]	68.7 ± 20.2 [54.3, 83.1]	85.8 ± 15.8 [75.2, 96.4]	87.9 ± 19.1 [75.1, 100.7]	90.0 ± 23.4 [74.3, 105.7]	Sex	14.8
LA ESV (mL)	66.2 ± 8.6 [60.0, 72.3]	68.1 ± 11.2 [60.0, 76.1]	66.4 ± 14.4 [56.2, 76.7]	93.3 ± 12.5 [85.0, 101.7]	95.4 ± 17.0 [84.0, 106.8]	93.6 ± 20.0 [80.2, 107.1]	Sex	533.7
LA ESV iBSA (mL/m^2^)	35.4 ± 5.5 [31.4, 39.3]	36.3 ± 6.6 [31.5, 41.1]	35.2 ± 6.8 [30.3, 40.0]	43.1 ± 5.3 [39.5, 46.7]	44.1 ± 7.3 [39.2, 49.0]	43.0 ± 8.2 [37.5, 48.5]	Sex	14.8
RA area (cm^2^)	15.0 ± 2.4 [13.3, 16.6]	15.6 ± 2.7 [13.6, 17.5]	15.9 ± 3.0 [13.8, 18.0]	18.1 ± 2.3 [16.6, 19.7]	19.5 ± 2.4 [17.9, 21.1]	20.2 ± 3.4 [17.9, 22.4]	Sex	80.0
RA ESV (mL)	42.0 ± 13.7 [32.2, 51.9]	44.9 ± 15.0 [34.1, 55.6]	45.3 ± 14.4 [35.0, 55.6]	50.4 ± 14.6 [40.6, 60.2]	59.9 ± 14.0 [50.5, 69.4]	64.9 ± 16.8 [53.6, 76.2]	Training + sex	7.6
RA ESV iBSA (mL/m^2^)	22.5 ± 7.6 [17.1, 27.9]	23.8 ± 7.7 [18.3, 29.3]	24.1 ± 7.4 [18.8, 29.5]	23.2 ± 6.2 [19.1, 27.4]	28.0 ± 7.8 [22.8, 33.3]	30.1 ± 8.3 [24.5, 35.6]	Training	0.9
RV mid-cavity diameter (cm)	2.5 ± 0.1 [2.4, 2.6]	2.5 ± 0.3 [2.3, 2.7]	2.6 ± 0.5 [2.2, 2.9]	2.7 ± 0.3 [2.5, 2.9]	2.9 ± 0.3 [2.7, 3.0]	2.7 ± 0.4 [2.5, 3.0]	Sex	1.7
RV basal diameter (cm)	3.2 ± 0.3 [3.0, 3.5]	3.4 ± 0.2 [3.3, 3.6]	3.5 ± 0.3 [3.3, 3.8]	3.8 ± 0.3 [3.6, 4.0]	4.1 ± 0.4 [3.8, 4.6]	3.9 ± 0.4 [3.7, 4.2]	Training + sex	921.9
RV *S′* (cm/s)	13.8 ± 1.4 [12.6, 15.1]	13.9 ± 1.7 [12.3, 15.4]	12.2 ± 0.8 [11.4, 13.0]	15.7 ± 1.6 [14.6, 16.8]	15.6 ± 2.2 [14.2, 17.1]	14.9 ± 2.2 [13.4, 16.4]	Training + sex	18.5
TAPSE (cm)	2.4 ± 0.3 [2.1, 2.7]	2.6 ± 0.7 [2.1, 3.1]	2.5 ± 0.3 [2.3, 2.8]	2.9 ± 0.4 [2.6, 3.2]	2.8 ± 0.3 [2.5, 3.0]	2.8 ± 0.3 [2.3, 2.8]	Sex	2.4
*E* (m/s)	0.81 ± 0.09 [0.75, 0.87]	0.85 ± 0.10 [0.78, 0.92]	0.79 ± 0.09 [0.73, 0.85]	0.85 ± 0.14 [0.74, 0.94]	0.79 ± 0.17 [0.67, 0.90]	0.82 ± 0.14 [0.71, 0.92]	Sex	0.5
*e′* (cm/s)	11.3 ± 1.0 [10.6, 12.0]	10.7 ± 1.5 [9.6, 11.7]	10.4 ± 1.3 [9.5, 11.3]	11.2 ± 1.0 [10.4, 11.9]	10.6 ± 1.6 [9.5, 11.7]	11.5 ± 1.1 [10.7, 12.3]	Training	0.6
*E*/*e′* (ratio)	5.8 ± 0.7 [3, 6.3]	6.2 ± 0.4 [5.9, 6.5]	5.8 ± 0.7 [5.4, 6.2]	6.2 ± 1.4 [5.1, 7.4]	5.4 ± 1.0 [4.6, 6.3]	5.9 ± 0.9 [5.1, 6.6]	Interaction	0.5
**2D speckle-tracking echocardiogram**								
2D GLS (%)	− 22.9 ± 2.1 [− 24.4, − 21.4]	− 22.2 ± 2.4 [− 23.9, − 20.5]	− 21.1 ± 1.8 [− 22.3, − 19.8]	− 21.8 ± 2.3 [− 23.3, − 20.3]	− 21.6 ± 2.4 [− 23.9, − 20.5]	− 23.0 ± 2.0 [− 24.4, − 21.7]	Sex	0.3
**3D and 3D speckle-tracking echocardiogram**								
3D LV Mass (g)	211.6 ± 20.4 [183.5, 239.5]	221.2 ± 20.6 [193.5, 249.8]	214.6 ± 20.9 [186.2, 243.4]	285.1 ± 20.4 [255.8, 311.8]	281.9 ± 20.7 [252.6, 308.9]	301.9 ± 20.9 [272.9, 330.2]	Interaction	2783.5
3D LV Mass iBSA (g/m^2^)	112.3 ± 7.9 [101.2, 123.4]	117.2 ± 8.0 [106.1, 128.6]	114.2 ± 6.6 [102.8, 125.6]	132.4 ± 7.9 [120.9, 143.2]	131.3 ± 8.0 [119.5, 142.1]	139.7 ± 6.6 [128.2, 151.1]	Interaction	252.3
3D LV Mass iLBM (g/kg)	3.9 ± 0.5 [3.5, 4.2]	–	4.0 ± 0.4 [3.8, 4.3]	3.9 ± 0.4 [3.6, 4.2]	–	4.1 ± 0.4 [3.8, 4.3]	Training	0.9
3D LV EDV (mL)	142.4 ± 21.3 [126.9, 157.8]	152.4 ± 21.6 [137.0, 167.8]	142.0 ± 15.6 [130.8, 153.1]	203.7 ± 26.8 [185.7, 221.7]	201.6 ± 32.6 [179.7, 223.5]	216.3 ± 36.0 [192.1, 240.5]	Sex	2789.6
3D GLS (%)	− 22.8 ± 2.4 [− 24.5, − 21.1]	− 22.2 ± 2.5 [− 24.1, − 20.4]	− 21.5 ± 1.9 [− 22.9, − 20.2]	− 22.3 ± 2.4 [− 23.8, − 20.7]	− 22.3 ± 3.1 [− 24.4, − 20.3]	− 23.9 ± 2.1 [− 25.3, − 22.5]	Sex	0.4
3D Global Strain (%)	− 34.9 ± 5.7 [− 39.0, − 30.8]	− 33.8 ± 2.6 [− 35.6, − 31.9]	− 33.2 ± 2.6 [− 35.1, − 31.3]	− 34.2 ± 3.5 [− 36.5, − 31.8]	− 34.9 ± 3.6 [− 37.3, − 32.5]	− 35.8 ± 3.2 [− 37.9, − 33.7]	Sex	0.5
3D GCS (%)	− 29.8 ± 6.6 [− 34.5, − 25.1]	− 28.0 ± 2.4 [− 29.7, − 26.2]	− 28.5 ± 2.5 [− 30.3, − 26.7]	− 28.0 ± 3.8 [− 30.6, − 25.5]	− 28.1 ± 4.5 [− 31.1, − 25.1]	− 28.7 ± 3.4 [− 31.0, − 26.5]	Sex	0.4
3D GRS (%)	44.4 ± 6.3 [39.9, 48.9]	42.3 ± 3.7 [39.6, 45.0]	41.7 ± 2.9 [39.6, 43.7]	42.0 ± 3.9 [39.3, 44.6]	41.7 ± 4.5 [38.7, 44.8]	43.6 ± 2.9 [41.7, 45.6]	Sex	0.4
3D Twist (°)	12.8 ± 4.8 [9.3, 16.2]	11.2 ± 4.1 [8.3, 14.2]	15.4 ± 3.4 [12.9, 17.8]	12.8 ± 6.6 [8.4, 17.2]	15.2 ± 5.2 [11.7, 18.7]	13.3 ± 6.3 [9.1, 17.6]	Sex	0.4
3D Torsion (°)	1.4 ± 0.5 [1.0, 1.7]	1.2 ± 0.5 [0.9, 1.5]	1.7 ± 0.4 [1.4, 1.9]	1.2 ± 0.7 [0.8, 1.7]	1.5 ± 0.5 [1.1, 1.8]	1.3 ± 0.6 [0.9, 1.7]	Sex	0.4

Best model: the repeated measures analysis of variance that best explains the data. Sex indicates a difference between males and females, training indicates a difference across the training season, Training + sex indicates independent effects of sex and training, interaction refers to an interaction between sex + training + sex × training

BF_10_: level of evidence 0–1: no effect, 1–3: anecdotal, 3–10: moderate, 10–30: strong, 30–100: very strong, > 100: extreme

*Early* early season, *Mid* mid-season, *Late* late season, *IVSd* interventricular septum in diastole, *LVIDd* left ventricular internal end-diastolic diameter, *LVIDs* left ventricular internal end-systolic diameter, *LVPWd* left ventricular posterior wall end diastole, *RWT* relative wall thickness, *E*/*e′* the ratio between early mitral inflow velocity and mitral annular early diastolic velocity, *LV* left ventricular, *EDV* end-diastolic volume, *ESV* end-systolic volume, *SV* stroke volume, *GLS* global longitudinal strain, *GCS* global circumferential strain, *GRS* global radial strain, *RA* right atrial, *LA* left atrial, *RV* right ventricle, *S′* systolic excursion velocity, *TAPSE* tricuspid annular plane systolic excursion, *iBSA* index to body surface area, *iLBM* index to lean body mass

An extremely strong interaction effect was evident in 3D absolute LVM (BF_10_ = 2783.5), suggesting that the differences between sexes changed across training blocks. In females, 3D LVM increased and peaked at MS (221.2 ± 20.6 g) following training Block 1 (ES to MS) and decreased after training Block 2 (MS to LS). In contrast, 3D LVM in males decreased modestly after training Block 1 (ES to MS), followed by an increase after training Block 2 (MS to LS), reaching a peak value of 301.9 ± 20.9 g at LS. When indexing 3D LVM for BSA, the evidence supporting the interaction remained extremely strong (BF_10_ = 252.3) and the direction of change with training was the same for each sex as non-indexed values. However, indexing for LBM eliminated the differences between sexes and with training (BF_10_ = 0.9).

For both 2D and 3D LV EDV, sex differences were supported by extremely strong evidence (BF_10_ = 105.6 and 2789.6, respectively) with females exhibiting smaller LV ventricular volume compared to males. This was reduced to moderate when 2D LV EDV was indexed for BSA (BF_10_ = 3.4). Sex differences were also very strong evident for stroke volume (BF_10_ = 54.0), favouring larger volume in males compared to females at all time points. A difference in sex and with training was observed for 2D LV ESV (BF_10_ = 536.9), whereby females had consistently smaller volume compared to males and all athletes demonstrated the lowest LV ESV at ES. Differences in ejection fraction were marginal (BF_10_ = 2.8).

Extremely strong evidence (BF_10_ = 129.9) for a sex difference, was evident for internal diameter of the LV during diastole (LVIDd); however, the sex difference was removed when indexed for BSA (BF_10_ = 0.7). Differences in sex and with training were observed in LVIDs, with extremely strong supporting evidence (BF_10_ = 619.9). Females had smaller internal diameters compared to males for both variables whilst LVIDs was smallest at ES in in both sexes. Right ventricular basal diameter was also best predicted by the model including both training and sex with extremely strong evidence in support of this (BF_10_ = 921.9). Males had larger RV diameter compared to females, whereas all athletes had the smallest RV basal diameter at ES compared to MS and LS. In contrast, there was only anecdotal evidence for a difference in sex for RV mid-cavity diameter (BF_10_ = 1.7). A moderate effect (BF_10_ = 18.5) for sex and with training was evident for RV *S′*, which was greater in males compared to females and the lowest in all athletes at LS. Differences between the sexes for TAPSE were only anecdotally supported (BF_10_ = 2.4).

There was extremely strong evidence supporting the sex differences in LVPWd (BF_10_ = 1833.0), with females presenting with thinner walls compared to males. Relative wall thickness was also smaller in females, which was supported by a model containing only sex (BF_10_ = 11.2). The variability in IVSd was supported by the model containing both training and sex with overwhelming evidence (BF_10_ = 11,044.2). Again, smaller absolute values were observed in females compared to males at all time points whilst IVSd was smallest at ES, irrespective of sex.

Differences between sexes in left atrial end-systolic volume were supported by extreme evidence (BF_10_ = 533.7), with males having increased volume compared to females. When indexed for BSA, sex was reduced to a strong effect (BF_10_ = 14.8). Differences between sexes in left atrial area and volume were supported by strong evidence (BF_10_ = 15.7 and BF_10_ = 14.8, respectively), with males demonstrating larger LA area and volume compared to females. Sex was again the best model to predict RA area with very strong evidence for RA area (BF_10_ = 80.0), favouring larger values in males compared to females. There was moderate evidence for meaningful effects of both sex and training on right atrial end-systolic volume (BF_10_ = 7.6), which was higher in males compared to females and increased throughout the season; however, these differences were not evident when indexing for BSA (BF_10_ = 0.9).

Bayes factors were < 1.0 across all strain variables including 2D and 3D GLS, twist and torsion, 3D global stain, 3D GCS, 3D GRS and some diastolic function variables including *E*, *e′* and *E*/*e′*. Thus, evidence supports the null hypothesis that these variables did not differ between the sexes or time points.

**Fig. 2:**
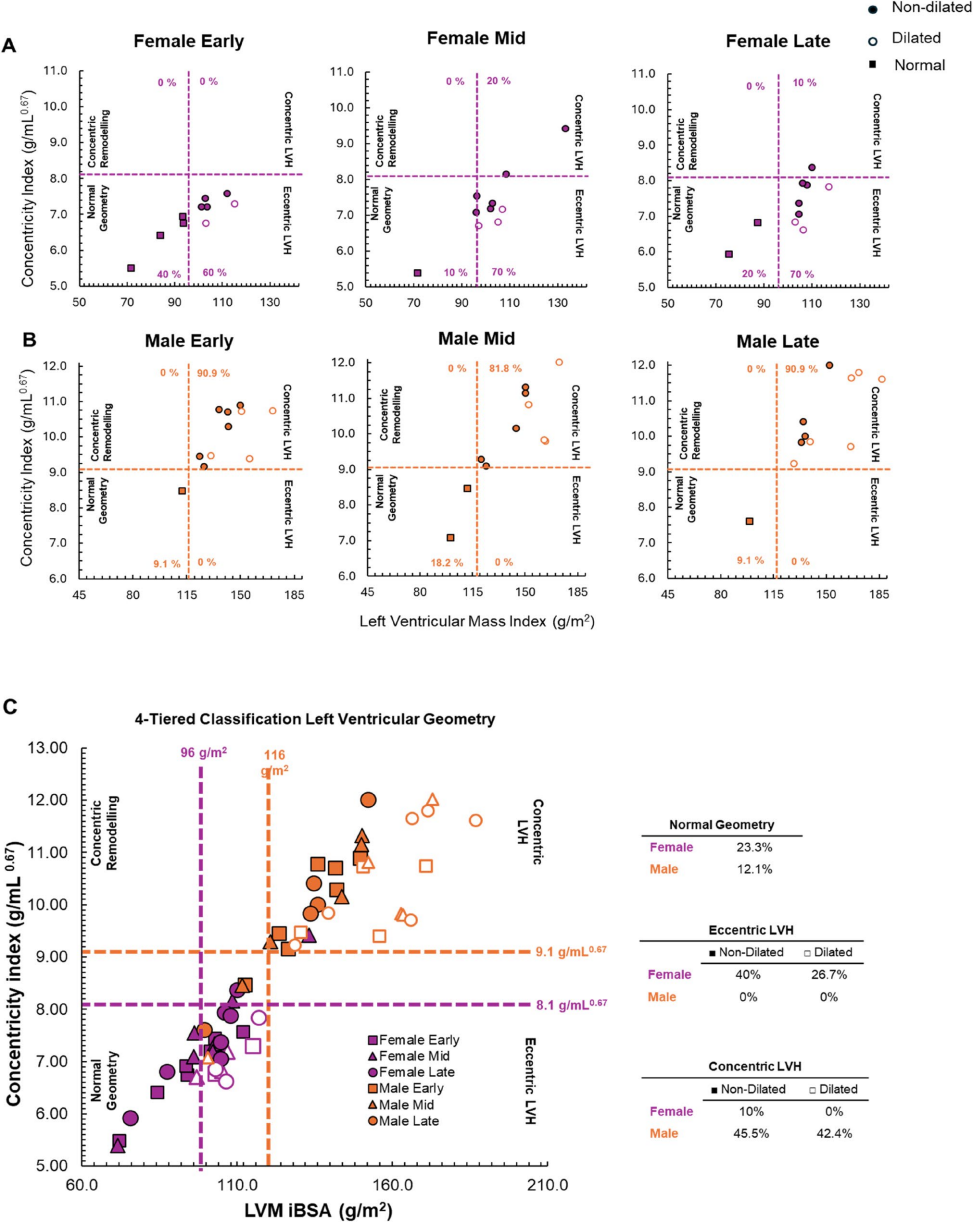
Left ventricular hypertrophy (LVH) classification across the 21-week competitive rowing season in elite females (Panel **A**, *n* = 10) and males (Panel **B**, *n* = 11), assessed at Early, Mid- and Late season. Panel **C** presents both sexes across all time points for direct comparison. Thresholds for 4-tier classification indicate sex-specific cut-offs; left ventricular mass index (females ≥ 96 g/m2; males ≥ 116 g/m2), concentricity index (left ventricular mass/end-diastolic volume; females ≥ 8.1 g/mL0.67; males ≥ 9.1 g/mL0.67 males). Dilated LVH was determined from left ventricular end-diastolic volume index (≥ 76 mL/m2 for both sexes)

Left ventricular hypertrophy classification and changes across the season are presented in Fig. 2. Across the study period, 23.3% of females and 12.2% of males were classified with normal geometry. Most females were classified with eccentric hypertrophy (40% non-dilated, 26.7% dilated) with 10% classified with concentric non-dilated hypertrophy. No males were classified with eccentric hypertrophy, with 45.5% classified with non-dilated concentric hypertrophy and 42.4% with dilated concentric hypertrophy.

### Training volume and intensity

Aggregated training volumes and intensities are presented in Table 3. There were no differences in the number of conditioning sessions between the sexes or training Blocks 1 and 2 (BF_10_ = 0.6). There was only marginal evidence that males and females differed in the number of resistance sessions across both training blocks (BF_10_ = 1.2). Evidence favoured the null hypothesis of no differences across sexes or training for the number of minutes spent in T1, T2 or T3 (all BF_10_ < 1.0). An interaction effect was evident for time spent in T4 and T5. The evidence supporting T4 variance was moderate (BF_10_ = 4.7) whilst T5 was very strong (BF_10_ = 32.8). Overall, males spent more time at higher intensities (in T4 and T5) compared to females during both training blocks. Females spent a similar amount of time in T4/T5 during each block, whilst males spent less time in T4/T5 during training Block 2 compared to Block 1.

**Table 3:** Training volume (average number of weekly conditioning and resistance sessions), and intensity (minutes spent in each heart rate training zone (T1-T5) per week) in elite female (*n* = 10) and male (*n* = 11) rowers across a competitive season (21 weeks)

	Female athletes (*n* = 10)		Male athletes (*n* = 11)		Best model	BF_10_
	Block 1 (Early Season–Mid-Season)	Block 2 (Mid-Season–Late Season)	Block 1 (Early Season–Mid-Season)	Block 2 (Mid-Season–Late Season)		
Number of conditioning sessions (sessions/week)	11.0 ± 3.4 [8.6, 13.5]	12.7 ± 2.7 [10.7, 14.6]	12.6 ± 3.2 [10.4, 14.7]	12.5 ± 4.2 [9.7, 15.3]	Training	0.6
Number of resistance sessions (sessions/week)	2.9 ± 1.1 [2.1, 3.7]	2.3 ± 0.6 [1.9, 2.6]	1.7 ± 1.3 [0.8, 2.6]	2.1 ± 0.9 [1.5, 2.7]	Sex	1.2
T1 (mins/week)	216.6 ± 84.5 [144.3, 288.4]	194.2 ± 53.9 [155.7, 232.8]	209.5 ± 84.5 [152.7, 266.2]	187.1 ± 89.7 [126.8, 247.4]	Training	0.6
T2 (mins/week)	164.9 ± 59.2 [122.5, 207.2]	168.2 ± 62.8 [123.4, 213.1]	186.4 ± 88.3 [127.0, 245.7]	176.4 ± 101.0 [105.3, 247.6]	Sex	0.6
T3, mins/week	68.0 ± 41.8 [38.1, 97.9]	65.6 ± 38.9 [37.9, 93.5]	61.1 ± 33.3 [38.7, 83.5]	58.2 ± 30.6 [37.7, 78.8]	Sex	0.5
T4 (mins/week)	14.0 ± 9.8 [7.0, 21.0]	14.2 ± 9.9 [7.1, 21.3]	27.3 ± 12.0 [19.3, 35.4]	19.9 ± 10.5 [12.8, 26.9]	Interaction	4.7
T5 (mins/week)	3.9 ± 3.5 [1.4, 6.4]	4.0 ± 3.7 [1.4, 6.6]	13.8 ± 9.9 [7.1, 20.4]	10.0 ± 9.0 [4.0, 16.1]	Interaction	32.8

Heart rate zones are relative to lactate thresholds 1 and 2 using a 5-zone model as follows; T1: between 50% of V̇O_2_peak and the midway point between 50% V̇O_2_peak and lactate threshold 1, T2: between the top of T1 and lactate threshold 1, T3: between lactate threshold 1 and 95% of lactate threshold 2, T4: between 95% and 102% lactate threshold 2, T5: above lactate threshold 2. Data is presented as (mean ± standard deviation [95% credible interval]) and the best model to explain the data from a Bayesian repeated measures of analysis and the Bayes factor (BF_10_) are reported

Heart rate was not captured during all sessions due to malfunction of the watch (e.g. batteries dying, placement incorrect, data not being fully recorded), session not being suitable for heart rate (e.g. swimming), or user error (e.g. athlete not wearing watch)

Best model: the repeated measures analysis of variance that best explains the data. Sex indicates a difference between males and females, training indicates a difference across the training season, Training + sex indicates independent effects of sex and training, interaction refers to an interaction between sex + training + sex × training

BF_10_: level of evidence 0–1: no effect, 1–3: anecdotal, 3–10: moderate, 10–30: strong, 30–100: very strong, > 100: extreme

*ES* early season, *MS* mid-season, *LS* late season, *LT* lactate threshold

### Menstrual status

Table 4 outlines all objectively measured hormone profiles for individual female athletes. Female athletes were a mix of hormonal contraceptive users (*n* = 6) and non-contraceptive users (*n* = 4). Of those who were not using hormonal contraception, one was classified as naturally menstruating with an average cycle length 26.0 ± 1.7 days and six reported periods during the study (Athlete 3). Two athletes were classified as oligomenorrhea due to cycle length > 35 days (Athletes 1 and 4) and one athlete was classified as likely having secondary amenorrhea due to the absence of periods for the duration of the study (Athlete 2); however, these observed menstrual irregularities were not diagnosed by a medical practitioner. Athletes using hormonal contraception were either using triphasic oral contraceptive (*n* = 4; *n* = 2: *LEVLEN® ED* [levonorgestrel, ethinylestradiol], *n* = 2: *Yasmin* [drospirenone, ethinylestradiol])) or long-acting reversible contraception; implantable device (*n* = 1: *Implanon NXT*®, Athlete 5) or intra-uterine device (*n* = 1, *Mirena* ®, Athlete 6). Two of the athletes changed their type of hormonal contraception, one ceased hormonal contraceptive use altogether between ES and MS (Athlete 9) and one athlete changed from combined oral contraceptive pill to a hormonal implant between MS and LS (Athlete 7, *Yasmin* to *Mirena®*).

**Table 4:** Menstrual cycle and hormone profiles of individual elite female rowers (n = 10) across a competitive season (21 weeks, measured at three time points: early, mid- and late season

Athlete	Menstrual status	Cycle length/length between bleeds, days ± SD	Number of periods/WB	Early season				Mid-season				Late season			
				Phase during testing	E2 pmol/L	P4 nmol/L	T nmol/L	Phase during testing	E2 pmol/L	P4 nmol/L	T nmol/L	Phase during testing	E2 pmol/L	P4 nmol/L	T nmol/L
1	NC	41.7 ± 18.4	3	Phase 4	140	10.0	< 1	Phase 3	720	31.0	1.0	Phase 1	110	< 1	0.9
2	NC	N/A	1	N/A				Phase 1	270	< 1	1.2	N/A	97.0	< 1	1.1
3	NC	26.0 ± 1.7	6	N/A				Phase 4	310	18.0	0.8	Phase 4	580	36.0	1.1
4	NC	43.0 ± 21.2	3	N/A				Phase 2	460	< 1	1.2	N/A	170	< 1	1.3
5	Implant	45.7 ± 35.8	4	N/A	110	< 1	0.6	WB	240	< 1	0.9	WB	140	< 1	0.7
6	IUD	N/A	1	N/A	170	< 1	1.0	N/A	1700	1.0	1.2	N/A	98	< 1	1.1
7	OCP/IUD^a^	36.3 ± 16.6	4	WB	< 40	< 1	0.7	AP	< 40	2.0	0.9	N/A	240	1.0	0.8
8	OCP	27.5 ± 9.5	5	AP	N/A			AP	< 40	< 1	0.6	AP	70	< 1	0.8
9	OCP/NC^b^	31.3 ± 8.0	5	AP	N/A			Phase 1	210	< 1	0.9	Phase 2	690	< 1	0.9
10	OCP	31.3 ± 22.2	4	AP	N/A			AP	< 40	< 1	0.7	AP	< 40	< 1	0.6

*NC* naturally cycling or not on hormonal contraception, *OCP* oral contraceptive pill, *Implant* hormonal contraceptive device implanted within the arm, *IUD* hormonal contraceptive intra-uterine device, *WB* withdrawal bleed, *AP* active oral contraceptive pills, *N/A* not available (blood sample unable to be taken or phase unable to be objectively identified), *E2* Oestradiol, *P4* Progesterone, *T* total testosterone

Phases classified using Elliot-Sale et al., 2021, *Sports Medicine, 51*(5), p. 854

^a^Changed between mid-season and late season

^b^Changed between early season and mid-season

## Discussion

This study measured echocardiographic-derived cardiac adaptation, performance outcomes and training volumes across a 21-week rowing season comparing young, highly trained elite female and male rowers. Our cardiac data presented here support previous works that recognise sex is an important modifier of cardiac morphology in athlete cohorts whereby females had lower 2D and 3D-derived cardiac mass and volumes, which persisted despite indexation, as well as reduced ventricular wall thicknesses, smaller biventricular internal diameters and RA area (D’Ascenzi et al. 2020; Kleinnibbelink et al. 2021). We also observed evidence for rowing training-specific adaptation in both sexes for interventricular septal thickness, LV end-systolic diameter, RV basal diameter and LV ESV. Interestingly, sex-specific response to training was evident for LVM when measured using 3D echocardiography, suggesting a difference in the direction of change with training in males and females.

Recent research has shown continued plasticity of elite athletes’ heart with continued training (Castillo et al. 2023; Forsythe et al. 2023; Kleinnibbelink et al. 2021). This is typically studied over extended blocks of time, typically from 3 to 9 months in duration however is often limited to a pre–post-assessment (Kleinnibbelink et al. 2021) rather than temporal, repeated measurements corresponding to key training blocks with specific prescription (e.g. general preparation vs race-specific). Others have focussed exclusively on responses in male athletes (Forsythe et al. 2023) or failed to present sex-disaggregated data (Wasfy et al. 2019), leaving a noticeable gap in female athlete-specific research. The present study aims to assess cardiac adaptations within a single season in a cohesive training group consisting of both male and female athletes, including detailed training exposure between sexes and with training. Whilst conditioning and resistance training exposure was generally the same between sexes, males spent more time (~ 17 min per week) at higher heart rates (T4 and T5) compared to females. We observed training-induced changes independent of sex in interventricular septal thickness, LV end-systolic diameter, RV basal diameter, RA ESV and LV ESV, with the values observed being the lowest at early season compared to both mid- and late season in all athletes. For RV *S′*, we observed the lowest value at late season compared to both early and mid-seasons. The amount of time in each heart rate zone has been shown to affect specific performance adaptations in rowing (Watts et al. 2024) highlighting that training intensity may influence specific cardiac adaptation. Whilst this coincides with a greater volume of time spent in the maximal aerobic training zone in males during Block 1, the potential training stimulus in females driving this adaptation is unclear. This is further emphasised by the largest increases in performance measures between early and mid-season in males, whilst females exhibited the highest V̇O_2_ peak at late season. However, there are limitations with capturing heart rate during high-intensity training with heart rate straps as they are less reliable at this intensity due to cardiac lag, often underestimating the time spent in higher heart rate zones, particularly with short interval bursts (Watts et al. 2024). Furthermore, relative intensity can vary between individuals, even with the same predicted HRmax or fitness. To compensate for this, we individualised the heart rate zones for each athlete based on their most recent V̇O_2_ peak data, together with lactate threshold assessment, and utilised a 5-zone model to assist in understanding the adaptation to training, rather than relying on age-predicted models alone (Watts et al. 2022). Previous literatures have shown either no changes in cardiac adaptation across a season (Forsythe et al. 2023), sex-specific adaptation (Kleinnibbelink et al. 2021), or chamber specific adaptation (Lakatos et al. 2020), accentuating that there is currently no widely accepted consensus. A comprehensive study by Marsh et al. (2021) suggested that untrained females may take longer to adapt to an endurance training stimulus compared to males, whilst others suggest females adapt quickly i.e. within 3 months, which plateaus with further training exposure (Howden et al. 2015). Whilst only anecdotal, our female rowers were younger and had a lower training age compared to males and may therefore still be yet to reach their ‘peak’ cardiac adaptation. Future studies should consider longitudinal evaluation of athletes commencing at a younger age to establish the association between chronic training exposure and cardiac response.

We observed meaningful sex distinctions favouring males for LV end-diastolic internal and posterior wall diameter, relative wall thickness, LV end-diastolic volume, LA area, LA end-diastolic volume and RA area. These findings support the growing literature reinforcing the notion that sex is a predictor of cardiac size in athletes and influences exercise-induced cardiac adaptation (Churchill et al. 2021; D’Ascenzi et al. 2020; Kleinnibbelink et al. 2021). We also found strong evidence for male sex effects for several physical characteristics, such as height, body mass, and BSA, which may partially explain the magnitude of cardiac size differences. Scaling for body size reduced the differences between sexes in all instances, in some variables scaling for lean body mass removed any sex effect altogether, highlighting the relevance of appropriate scaling for cardiac variables (St. Pierre et al. 2022).

Unsurprisingly, male rowers demonstrated larger LVM compared to females when measured using both 2D and 3D echocardiography, including when indexing for BSA. Indexing to LBM reduced the difference between sexes suggesting adaptation of the LV corresponds to muscular adaptation in the rest of the body (Kooreman et al. 2019). However, a moderate sex difference remained, emphasising that skeletal muscle mass is not the only driver of cardiac mass in elite rowers. Of note, when LVM was measured using 3D echocardiography, we observed an interaction effect suggesting a sex-specific response to a similar training volume (conditioning sessions per week). Specifically, LVM increased in both females and males from early to late season by 1.4 and 5.9%, respectively. However, the direction and the magnitude of change differed by sex and training block; females experienced the greatest gain in cardiac mass during training Block 1 (+ 4.6%) whilst males demonstrated a concomitant decrease (− 1.1%). After training Block 2 however, LVM in females decreased by 3.1% whilst males gained an additional 7.1% of LVM compared to early season. This observation highlights two relevant paradigms when assessing change in cardiac mass in athletes: first, assessing the cardiac parameters using 2D and 3D echocardiography and second, the ability to accurately quantify change over time. Indeed, 2D-derived cardiac mass has reduced accuracy compared to cardiac MRI (Spence et al. 2013); however, 3D-derived cardiac mass and volume have a strong correlation with MRI and can accurately detect changes over time (De Bosscher et al. 2023). Although MRI is considered the ‘gold standard’ criterion measurement for cardiac morphological assessment, echocardiography is more widely accessible and clinically utilised with its application recommended as a first-line diagnostic approach to differentiate normal from pathological phenotypes and is indeed mandated by some professional sporting federations including FIFA (D’Andrea et al. 2021; Pelliccia et al. 2018). Further to this, embedding the practice of 3D echocardiography may provide a more accurate and reproducible alternative to MRI in the clinical evaluation of athletes when assessing change over time.

Similar to previous findings (Finocchiaro et al. 2017), this study observed a higher proportion of females classified with eccentric hypertrophy compared to males, who more frequently demonstrated concentric hypertrophy. In fact, no male rowers in this study were classified with eccentric hypertrophy. In contrast to studies that classify athlete LV hypertrophy, we assessed changes in LV hypertrophy across a season, which shifted in both the males and females, with the general trend going towards the right (Fig. 2), moving away from normal geometry and favouring eccentric and concentric phenotypes. Brown et al. (2020) found that 33% of elite male cyclists had eccentric hypertrophy compared to only 3% of sub-elite cyclists, suggesting athlete calibre has an impact on the magnitude of adaptation, which could explain the shift in classification across the season. With the distinction between benign and pathological LV hypertrophy being an important clinical diagnosis, consideration of the level of athlete and time point of testing within the season is crucial. Furthermore, the methodological approach used to classify cardiac phenotype can also influence the classification (McGregor-Cheers et al. 2023) such that using the four-tier classification approach (concentricity index in lieu of relative wall thickness) can better define LV geometry, specifically in athletes (Brown et al. 2020). Variation within the literature of two-tier and four-tier methods highlights a need for consensus to allow comparison across studies and clarity for clinical assessment.

This study found no change in cardiac mechanics across the season, nor any effect of sex, in elite rowers. Studies in untrained males (Oxborough et al. 2019) and comparisons between male athletes to controls (Beaumont et al. 2017) observed some training-induced changes in STE-derived cardiac mechanics variables. Specifically, after 24 weeks of endurance and resistance exercise training, peak basal rotation increased in previously sedentary males (Oxborough et al. 2019) whilst meta-analytic data showed no difference in basal rotation between male endurance athletes and controls, despite reduced twist and apical rotation (Beaumont et al. 2017). In female team-sport athletes compared to non-athletic female controls, there were no differences in STE-derived global longitudinal, radial or circumferential strain between groups (Zacher et al. 2020). Taken together, these findings imply that subtle differences in cardiac mechanics may result from training, with any differences limited to males. However, owing to a dearth of female athlete-specific STE data, further evaluation of large, heterogeneous female athlete cohorts should be interrogated to confirm whether any female-specific training-induced changes in cardiac mechanics are present. We also observed no effects for LV diastolic function (*E*, *e′*, *E*/*e′*) and anecdotal evidence supporting sex effects for both TAPSE and ejection fraction, suggesting that male and female athlete hearts function similarly despite the differences in size. This is in contrast to previous literature that has observed sex differences in diastolic function, specifically significantly lower *E*/*e′* in male versus female Olympic athletes (D’Ascenzi et al. 2020) whilst Kleinnibbelink et al. reported lower TAPSE and higher* E* velocity in female Olympic-calibre rowers (Kleinnibbelink et al. 2021). In our athletes, the preservation of function, despite morphological change in our athletes, may relate to the existing trained status of athletes (Oxborough et al. 2019) or the time course of change in functional compared to morphology adaptation (Kleinnibbelink et al. 2021; Weiner et al. 2015).

A major strength of the current study is the inclusion of detailed menstrual status data for all female athletes, which addresses the importance of data transparency and accurate methodological reporting in female populations (Elliott-Sale et al. 2021). Whilst this study did not specifically control for menstrual cycle phase or hormonal contraception use and is therefore unable to report on potential endocrine effects on exercise-induced adaptation of female athletes, we have continued to uphold scientific rigour with detailed reporting to enable future analyses to draw meaningful conclusions. Studying elite female athletic populations is inherently challenging with variability in contraception use, menstrual cycle regularity and in some cases, menstrual dysfunction. Several athletes in our study presented with menstrual irregularities that were not otherwise detected, underlining the importance of menstrual status monitoring in elite athlete cohorts for optimal health status.

Although the study sample size is limited, we included all rowing athletes classified as highly trained/national level or above and our sample captured most of the athletes within the immediate geographical area. The use of Bayesian statistics also accounts for the small sample size, providing a more trustworthy analysis of the results with the current sample. We did not examine blood volume and its potential impact on cardiac adaptation within this study as the primary aim of was focussed on the effects of exercise. Future research should consider including blood volume measurements to provide a comprehensive understanding of all factors potentially influencing cardiovascular adaptation. Lastly, the aggregated training data presented here were limited to time spent in respective HR zones and number of sessions per week and it is acknowledged there is a need to better quantify training load in rowing athletes to recognise and interrogate the multimodal nature of the training stimulus (Watts et al. 2024).

## Conclusion

We confirmed the presence of a sex-specific cardiac phenotype in young highly trained rowers of similar age and rowing-specific training history, whereby females had smaller cardiac dimensions which persisted despite accounting for body size, echoing previous literature (D’Ascenzi et al. 2020; Kleinnibbelink et al. 2021). Our study assessed rowers repeatedly over the course of a full season, accounting for the variation in periodised training blocks, and resulting training stimulus. We observed that whilst males spent a greater proportion of time training at a maximal heart rate overall, this decreased in the latter part of the season. Exercise-induced cardiac adaptation was evident for several echocardiographic-derived variables throughout the season, including cardiac volume, internal diameter, wall thickness as well as left atrial and right ventricular dimensions. Sex-specific adaptations were observed for LVM when measured using 3D echocardiography, highlighting the accuracy and reliability of this methodology for detecting training-induced change in athletes (De Bosscher et al. 2023). Future research should emphasise longer-term observation of elite-level athletes, for example, multi-year training cycles. Combining this with repeated measures aligned to key training and competition phases and detailed analysis of training exposure, will enable the quantification of a dose–response relationship between exercise exposure and resultant cardiac adaptation in both female and male athletes.

## Supplementary Information

Below is the link to the electronic supplementary material.

Supplementary file1 (DOCX 74 KB)

## Data Availability

Data is available on request.

## Abbreviations

2D: Two-dimensional
3D: Three-dimensional
BF_10_: Bayes factor
BSA: Body surface area
DXA: Dual-energy absorptiometry
*E*: Peak early mitral inflow velocity
*e′*: Mitral annular early diastolic velocity
EDV: End-diastolic volume
ES: Early season
ESV: End-systolic volume
GCS: Global circumferential strain
GLS: Global longitudinal strain
GRS: Global radial strain
IVSd: Interventricular septum end diastole
LA: Left atrium/ left atrial
LBM: Lean body mass
LS: Late season
LV: Left ventricle / ventricular
LVIDd: Left ventricular internal dimension diastole
LVIDs: Left ventricular internal dimension systole
LVPWd: Left ventricular posterior wall diastole
MS: Mid-season
RA: Right atrium / right atrial
RV: Right ventricle /ventricular
RV * S′*: Peak systolic velocity of the tricuspid annulus
RWT: Relative wall thickness
STE: Speckle-tracking echocardiography
SV: Stroke volume
TAPSE: Tricuspid annular plane systolic excursion
V̇O_2_: Oxygen consumption
V̇O_2_peak: Peak oxygen consumption
