# Supplementary material for: The effects of training and sex on cardiac adaptation in elite rowers across a competitive season
Source: Eur J Appl Physiol. 2025 Jul 25;126(1):397–412. doi: 10.1007/s00421-025-05897-w (PMC12881169; doi:10.1007/s00421-025-05897-w)
Supplement: Supplementary file 1 — Supplementary file1 (DOCX 74 KB) [file 421_2025_5897_MOESM1_ESM.docx]

**Supplementary materials**

**The effects of training and sex on cardiac adaptation in elite rowers across a competitive season.**

**Authors: Henley-Martin, Brade, Riddell, Watts, Maiorana, Collis, Green, Naylor, Binnie, and Spence**

Corresponding author: Angela L Spence, PhD

Curtin School of Allied Health,

Curtin University

GPO U1987, Perth, Australia 6845

Western Australia, Australia

Tel: +61 8 9266 3620

angela.spence@curtin.edu.au

**Supplementary Table 1** Bayesian factor for all models of a repeated measures analysis of variance for participant characteristics, cardiorespiratory (12-lead ECG determined heart rate), performance measures (peak exercise heart rate and oxygen consumption) and dual-energy absorptiometry derived anthropometric measure in elite female (n = 10) and male (n = 11) rowers across a competitive season (21 weeks), measured at three time-points: early-, mid- and late-season

|  | Bayes Factor (BF_10_) | | | |
| --- | --- | --- | --- | --- |
|  | Sex | Training | Training + sex | Interaction |
| Mass, kg | **44.6** | 0.2 | 9.7 | 2.3 |
| BSA, m^2^ | **34.2** | 0.2 | 7.3 | 1.8 |
| Total body fat, % | **31.6** | 0.3 | 10.8 | 5.0 |
| Lean body mass, % | **43.5** | 0.4 | 15.3 | 7.4 |
| Resting HR, bpm | 0.9 | 7.9 | **7.4** | 2.0 |
| Maximum HR, bpm | **0.6** | 0.4 | 0.2 | 0.1 |
| V̇O_2_peak, L.min^-1^ | 197706.4 | 9.9 | **1390000** | 631466.4 |
| V̇O_2_peak, mL kg^-1^ min^-1^ | 76.9 | 14.1 | 928.2 | **1258.2** |

BSA: body surface area, BPM: beats per minute, V̇O_2_peak: peak oxygen consumption

Sex indicates a difference between males and females, training indicates a difference across the training season, Training + sex indicates independent effects of sex and training, interaction refers to an interaction between sex + training + sex × training

BF_10_: level of evidence 0-1: no effect, 1-3: anecdotal, 3-10: moderate, 10-30: strong, 30-100: very strong, >100: extreme, the bolded number indicates the best model to explain the data.

**Supplementary Table 2** Bayesian factor for all models of a repeated measures analysis of variance for echocardiographic derived variables in elite female (n=10) and male (n=11) rowers across a competitive season (21 weeks), measured at three time-points: early-, mid- and late-season

|  | Bayes Factor (BF_10_) | | | |
| --- | --- | --- | --- | --- |
|  | Sex | Training | Training + sex | Interaction |
| LV Mass, g | **15724.5** | 0.6 | 9675.5 | 3742.8 |
| LV Mass iBSA, g/m^2^ | **772.0** | 0.6 | 458.2 | 134.6 |
| LV mass iLBM, g/kg | **5.2** | 1.0 | 5.0 | 1.9 |
| LV EDV, mL | **105.6** | 0.3 | 35.1 | 19.3 |
| LV EDV iBSA, mL/m^2^ | **1.0** | 0.2 | 0.2 | 0.1 |
| ESV, mL | 113.7 | 5.2 | **536.9** | 162.0 |
| SV, mL | **54.0** | 0.2 | 9.4 | 6.6 |
| Ejection fraction, % | 1.7 | 1.6 | **2.8** | 1.4 |
| Concentricity index, g/mL0.67 | **17683.5** | 0.4 | 7838.0 | 2881.2 |
| LVIDd, mm | **129.9** | 0.1 | 18.8 | 9.2 |
| LVIDd/BSA, mm/m^2^ | **0.7** | 0.1 | 0.1 | 0.0 |
| LVIDs, mm | 34.0 | 19.7 | **619.9** | 209.4 |
| IVSd, mm | 10983.1 | 1.0 | **11044.2** | 4740.8 |
| LVPWd, mm | **1833.0** | 0.2 | 422.0 | 142.6 |
| RWT, cm | **11.2** | 0.2 | 2.2 | 2.5 |
| LA area, cm^2^ | **15.7** | 0.2 | 3.1 | 0.8 |
| LA volume, mL | **14.8** | 0.1 | 2.2 | 0.5 |
| LA ESV, mL | **533.7** | 0.2 | 79.4 | 16.3 |
| LA ESV iBSA, mL/m^2^ | **14.8** | 0.2 | 2.4 | 0.5 |
| RA area, cm^2^ | **80.0** | 0.7 | 63.0 | 16.1 |
| RA ESV, mL | 6.3 | 1.1 | **7.6** | 3.3 |
| RA ESV iBSA, mL/m^2^ | 0.8 | **0.9** | 0.7 | 0.3 |
| RV mid-cavity diameter, cm | **1.7** | 0.2 | 0.3 | 0.1 |
| RV basal diameter, cm | 155.1 | 5.3 | **921.9** | 503.2 |
| RV S’, cm/s | 6.7 | 2.6 | **18.5** | 5.7 |
| TAPSE, cm | **2.4** | 0.2 | 0.3 | 0.1 |
| E, m/s | **0.5** | 0.2 | 0.1 | 0.1 |
| e’, cm/s | 0.5 | **0.6** | 0.3 | 0.5 |
| E/e’ | 0.5 | 0.2 | 0.1 | **0.5** |
| 2D speckle-tracking echocardiogram | | | | |
| GLS, % | **0.3** | 0.2 | 0.1 | 0.1 |
|  |  |  |  |  |
|  |  |  |  |  |
| 3D echocardiogram and 3D speckle-tracking echocardiogram | | | | |
| 3D LV Mass, g | 2473.6 | 0.5 | 1185.4 | **2783.5** |
| 3D LV Mass iBSA, g/m^2^ | 242.0 | 0.5 | 128.7 | **252.3** |
| 3D LV Mass iLBM, g/kg | 0.4 | **0.9** | 0.4 | 0.2 |
| 3D EDV, mL | **2789.6** | 0.2 | 557.2 | 677.0 |
| 3D GLS, % | **0.4** | 0.2 | 0.1 | 0.1 |
| 3D Global Strain, % | **0.5** | 0.1 | 0.1 | 0.03 |
| 3D GCS, % | **0.4** | 0.2 | 0.1 | 0.01 |
| 3D GRS, % | **0.4** | 0.2 | 0.1 | 0.1 |
| 3D Twist, deg | **0.4** | 0.2 | 0.1 | 0.1 |
| 3D Torsion, deg | **0.4** | 0.2 | 0.1 | 0.1 |

IVSd: interventricular septum in diastole, LVIDd: left ventricular internal end-diastolic diameter, LVIDs: left ventricular internal end-systolic diameter, LVPWd: left ventricular posterior wall end diastole, RWT: relative wall thickness, E/e’: the ratio between early mitral inflow velocity and mitral annular early diastolic velocity, LV: left ventricular, EDV: end diastolic volume, ESV: end systolic volume, SV: stroke volume, GLS: global longitudinal strain, GCS: global circumferential strain, GRS: global radial strain, RA: right atrial, LA: left atrial, RV: right ventricle, S’: systolic excursion velocity, TAPSE: tricuspid annular plane systolic excursion, iBSA: index to body surface area, iLBM: index to lean body mass

Sex indicates a difference between males and females, training indicates a difference across the training season, Training + sex indicates independent effects of sex and training, interaction refers to an interaction between sex + training + sex × training

BF_10_: level of evidence 0-1: no effect, 1-3: anecdotal, 3-10: moderate, 10-30: strong, 30-100: very strong, >100: extreme, the bolded number indicates the best model to explain the data

**Supplementary Table 3** Bayesian factor for all models of a repeated measures analysis of variance for training volume (average number of weekly conditioning and resistance sessions per week), and intensity (minutes spent in each training zone per week) in elite female (n = 10) and male (n = 11) rowers across a competitive season (21 weeks). Heart rate zones are relative to lactate thresholds 1 and 2 using a 5-zone model as follows; T1: between 50% of V̇O_2_peak and the midway point between 50% V̇O_2_peak and lactate threshold 1, T2: between the top of T1 and lactate threshold 1, T3: between lactate threshold 1 and 95% of lactate threshold 2, T4: between 95-102% lactate threshold 2, T5: above lactate threshold 2

|  | Bayes Factor (BF_10_) | | | |
| --- | --- | --- | --- | --- |
|  | Sex | Training | Training + sex | Interaction |
| Number of conditioning sessions, sessions/ week | **0.6** | 0.6 | 0.3 | 0.3 |
| Number of resistance sessions, sessions/ week | **1.2** | 0.3 | 0.4 | 0.7 |
| T1, mins/ week | **0.5** | 0.6 | 0.3 | 0.1 |
| T2, mins/ week | **0.6** | 0.3 | 0.2 | 0.1 |
| T3, mins/ week | **0.5** | 0.3 | 0.2 | 0.1 |
| T4, mins/ week | 2.0 | 1.2 | 2.6 | **4.6** |
| T5, mins/ week | 2.7 | 2.5 | 5.6 | **32.9** |

Note: heart rate was not captured during all sessions due to malfunction of the watch (e.g. batteries dying, placement incorrect, data not being fully recorded), session not being suitable for heart rate (e.g. swimming), or user error (e.g. athlete not wearing watch)

Sex indicates a difference between males and females, training indicates a difference across the training season, Training + sex indicates independent effects of sex and training, interaction refers to an interaction between sex + training + sex × training

BF_10_: level of evidence 0-1: no effect, 1-3: anecdotal, 3-10: moderate, 10-30: strong, 30-100: very strong, >100: extreme, the bolded number indicates the best model to explain the data
